# The Interplay Between Brain Vascular Calcification and Microglia

**DOI:** 10.3389/fnagi.2022.848495

**Published:** 2022-03-02

**Authors:** Upasana Maheshwari, Sheng-Fu Huang, Sucheta Sridhar, Annika Keller

**Affiliations:** ^1^Department of Neurosurgery, Clinical Neuroscience Center, Zürich University Hospital, University of Zürich, Zurich, Switzerland; ^2^Neuroscience Center Zürich, University of Zürich and ETH Zürich, Zurich, Switzerland

**Keywords:** astrocyte, cerebrovascular calcification, ectopic calcification, microglia, mouse model, pericyte, primary familial brain calcification, vascular aging

## Abstract

Vascular calcifications are characterized by the ectopic deposition of calcium and phosphate in the vascular lumen or wall. They are a common finding in computed tomography scans or during autopsy and are often directly related to a pathological condition. While the pathogenesis and functional consequences of vascular calcifications have been intensively studied in some peripheral organs, vascular calcification, and its pathogenesis in the central nervous system is poorly characterized and understood. Here, we review the occurrence of vessel calcifications in the brain in the context of aging and various brain diseases. We discuss the pathomechanism of brain vascular calcification in primary familial brain calcification as an example of brain vessel calcification. A particular focus is the response of microglia to the vessel calcification in the brain and their role in the clearance of calcifications.

## Introduction

Vascular calcification associated with vascular aging is prevalent in the elderly population. The presence of calcifications in blood vessels leads to vascular stiffness, loss of elasticity and decreased compliance causing impaired cardiovascular function and potential end-organ damage (Pescatore et al., 2019). Cerebral vascular calcification is considered a predictor of cardiovascular events such as heart attack as well as stroke (Rennenberg et al., 2009; Hermann et al., 2013). In addition, vascular calcification is a prevalent complication of diseases such as chronic kidney disease and diabetes and is associated with significant morbidity and mortality (Lanzer et al., 2021). Calcification of vessels also occurs in several genetic disorders or due to the systemic imbalance of phosphate metabolism (Takashi and Fukumoto, 2020; Rutsch et al., 2021).

**FIGURE 1:**
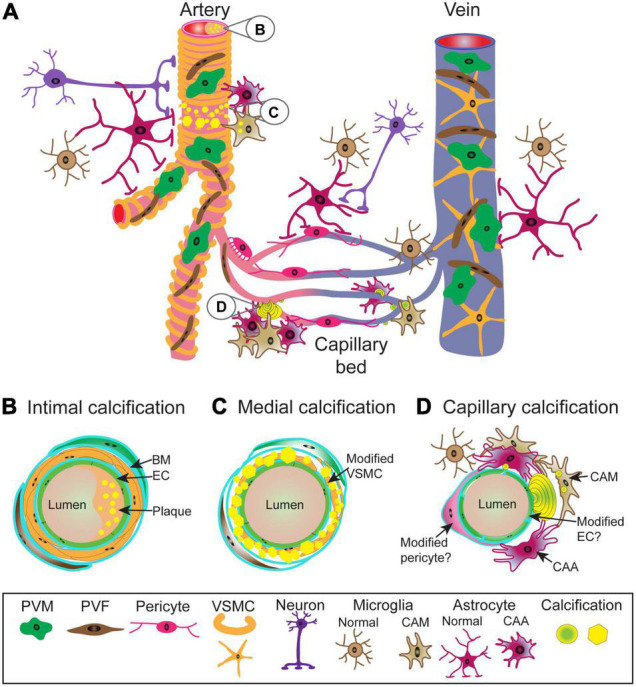
Schematic representation of vascular calcifications along the central nervous system (CNS) vascular network. Cellular and structural diversity of the vascular network along the arterio-venous axis **(A)**. In arteries, lumen forming endothelial cells (EC) are separated from circumferentially wrapped vascular smooth muscle cells (VSMC) by a basement membrane (BM). Capillary endothelial cells are covered by pericytes (PC) with a prominent cell body and slender processes with which they share the basement membrane and are in direct contact. Capillaries transition into veins which have an incomplete mural cell coverage. The entire vasculature is covered by astrocyte end-feet and occasionally innervated by nerve fibers. Between VSMC layer and astrocyte end-feet reside perivascular fibroblasts (PVF) and perivascular macrophages (PVM). Arterial calcifications can be intimal **(B)**, resulting from atherosclerotic plaque in the lumen, as well as medial **(C)**, due to phenotypic alterations of VSMCs leading to mineralization of extracellular matrix. It is not known to what extent PVM and PVF become modified and contribute to the pathophysiology. Capillary calcifications **(D)** are characterized by the appearance of mineralized “beads” on the capillary wall, which protrude into brain parenchyma. While it is known that capillary calcifications elicit strong glial reactions, changes in pericytes and endothelial cells in calcified capillaries are poorly understood. Calcifications are depicted as yellow spheres or polygons. BM, basement membrane; CAA, calcification-associated astrocyte; CAM, calcification-associated microglia; EC, endothelium; PVF, perivascular fibroblast; PVM, perivascular macrophage; VSMC, vascular smooth muscle cell.

Vasculature is divided into distinct zones based on their cellular and acellular composition, and function. Arteries are composed of lumen forming endothelial cells (*tunica intima*) separated from vascular smooth muscle cells (VSMC) (*tunica media*) by a basement membrane. Veins have incomplete VSMC coverage. Capillaries, the predominant vessel type in the brain, are positioned between arteries and veins. Capillaries are composed of endothelial cells and pericytes. In addition, the perivascular space along arteries and veins between the VSMC layer and astrocyte end-feet, which cover the entire vascular tree, contains several cell types, including perivascular macrophages and fibroblasts (Figure 1A).

Vascular calcifications can be distributed from the intimal layer (e.g., atherosclerosis) (Figure 1B) to the medial layer (Figure 1C) of the arterial wall, affecting different large and small arterial beds or capillaries (Figure 1D).

Unlike peripheral vascular calcification, intracranial calcifications (vascular or parenchymal) are less frequently reported and relatively poorly characterized. Intracranial calcifications are a common incidental finding in computed tomography (CT) imaging in the general population (Deng et al., 2015) and at autopsy. In addition to aging, brain calcifications occur due to metabolic alterations (e.g., chronic kidney disease, thyroid hormone imbalance), infections (e.g., viral encephalitis, neurocysticercosis, neurocryptococcosis), toxic injury (e.g., carbon monoxide poisoning, radio- and chemotherapy), genetic disorders (e.g., neurofibromatosis, tuberous sclerosis, primary familial brain calcification), brain tumors (e.g., oligodendrogliomas, meningiomas) as well as defective vascular morphogenesis during development (e.g., cavernomas, Sturge-Weber syndrome) (Nash et al., 2004; De La Torre et al., 2018; Donzuso et al., 2019; Saade et al., 2019). Parenchymal calcifications in the pineal gland, also referred to as “brain sand,” are even used as an anatomical landmark in radiographic studies (Vígh et al., 1998). The pathogenic mechanisms involved in brain calcifications in these diverse conditions are not well-understood, except in the case of systemic calcium and phosphate imbalances accompanying diseases such as kidney failure, hypo- or hyperparathyroidism. Often, while reported, intracranial calcifications are not clearly distinguished as parenchymal, vascular or both. Nevertheless, intracranial vessel-associated calcifications have been reported to accompany sporadic and familial neurodegenerative [e.g., Parkinson’s disease, Alzheimer’s disease (AD)] and neuroinflammatory diseases (e.g., type I interferonopathies) (Fujita et al., 2003; Livingston et al., 2013; Ukai and Kosaka, 2016; Donzuso et al., 2019; Saade et al., 2019).

In children, the presence of intracranial calcifications is almost always associated with underlying pathology (Goncalves et al., 2020a,b), whereas in adults, these are viewed as part of the normal aging process. However, recent studies have shown that intracranial calcifications are strong predictors of adverse clinical outcomes (Bartstra et al., 2020). We, therefore, believe that a detailed description of intracranial vascular calcification and related pathomechanism can potentially assist in early prognosis and further our understanding for better therapeutic targeting of brain diseases.

In this review, we describe brain vascular calcifications with a focus on possible underlying pathomechanism and the role of microglia as modifiers of brain vascular calcification. We primarily focus on the pathophysiology of vascular calcification occurring in a neuropsychiatric disease – primary familial brain calcification (PFBC).

## Bone in the Brain—An Overview of Cerebrovascular Calcifications

Calcification of large arteries such as the internal carotid artery, intracranial vertebrobasilar artery, middle cerebral artery, circle of Willis, as well as calcifications in the hippocampus and basal ganglia, are easily observed on CT images (Deng et al., 2015; Bartstra et al., 2020). It is estimated that 30% of aged individuals have brain calcifications (Nicolas et al., 2013a). However, due to relatively low resolution of CT imaging, additional histopathological analyses are required to identify calcification of microvasculature. Few case studies in aged human subjects have shown that both the calcification of the hippocampus and basal ganglia are vascular (Peters et al., 2018; de Brouwer et al., 2021). In the aged hippocampus, arteries, pre-capillaries and capillaries were calcified (Peters et al., 2018). Similar calcification of hippocampal vessels has been reported in AD patients (Wegiel et al., 2002; Schober et al., 2021). A recent study showed that the tunica media of arterioles in the globus pallidus is calcified in aged individuals, confirmed both histologically and on CT. Calcification of the vascular tree showed a distribution pattern, starting in the ventral striatopallidum spreading posterolaterally into the external half of the globus pallidus (de Brouwer et al., 2021). There is differential involvement of the vascular tree (artery, capillary), which might be dependent on the anatomical region and underlying disease. For example, previous histopathological studies detected arterial calcification in aged subjects and AD patients compared to capillary bed calcification in the globus pallidus in Down syndrome patients (Wegiel et al., 2002; Schober et al., 2021).

## Pathogenesis of Vascular Calcification

In bone, hydroxyapatite or carbonated hydroxyapatite crystals are deposited in the extracellular matrix consisting mainly of collagen I fibers (Arnold et al., 2021). Mineral formation also depends on the presence of metabolic ions (citrate, lactate, carbonate) and proteins as well as glycans that can modify the mineralization process by stabilizing crystals or preventing crystal growth (Young, 2003; Davies et al., 2014). Bone consists of three cell types—(i) osteoblasts- tissue mineralizing cells, (ii) osteoclasts- mineralized tissue resorbing cells, and (iii) osteocytes- long-lived cells located deep inside the bone matrix (Salhotra et al., 2020). Ectopic calcification of the vessel wall is thought to be an active process that resembles the formation of bone with hardening of the tissue initiated by nucleation of calcium phosphate in a permissive matrix. Hydroxyapatite and carbonated hydroxyapatite are the most abundant calcium phosphate phases in both physiological and pathological calcification. Although the primary cause of vascular calcification initiation might be different, it leads to an osteogenic environment. After initiation, osteoblast- and osteoclast-like cells, and bone matrix proteins can be detected in these mineralized structures. Transcriptional programs and signaling pathways involved in normal bone formation are also identified along the calcifying vessel (Chen Y. et al., 2021). In intimal calcification, calcium phosphate deposition occurs in so-called atherosclerotic plaques residing inside the blood vessel lumen. The extent of plaque calcification correlates with plaque stability (Motoyama et al., 2007). Calcification of the middle layer of the blood vessel wall (medial calcification) is associated with aging, diabetes, kidney disease, and hereditary vascular calcification diseases. Although the deposition of hydroxyapatite is a common feature between intimal and medial calcification, initiation and propagation mechanisms differ (Lanzer et al., 2014). The processes that trigger atherosclerotic plaque formation, calcification and the role of innate immunity in these processes are not discussed in this review.

The prevalent view is that medial calcification is accompanied by a phenotypic change of resident vascular smooth muscle cells into cells that promote mineralization. Vascular calcification can be initiated by several triggers such as cellular senescence, inflammation, loss of anti-calcifying proteins and imbalance in extracellular phosphate (Pi) and pyrophosphate (PPi) levels (Pescatore et al., 2019). Cellular senescence and inflammation have been shown to induce trans-differentiation of human primary VSMC into osteoblast-like cells capable of mineralizing the matrix (Nakano-Kurimoto et al., 2009; Sorokin et al., 2020). In addition to VSMC, circulating progenitor cells, mesenchymal stem cells, endothelial cells, and fibroblasts have been shown to share the potential to trans-differentiate into osteoblast-like cells in mouse models of atherosclerosis (Jiang et al., 2021). How can extracellular matrix calcification be controlled by osteoblast and osteoblast-like cells? Poly(ADP)-ribose (PAR) generated in response to cell damage signaling (either physiological by osteoclasts or pathological as in VSMC) is involved in matrix calcification (Pescatore et al., 2019). A recent study in mice reported the VSMC and osteoblasts deposit PAR-calcium, which is localized in bone collagen fibrils at the site of formation of calcium phosphate crystals during bone formation (Chow et al., 2014), into the extracellular matrix in response to vascular calcification. This process likely provides calcium for the initiation of calcium-phosphate crystals (Muller et al., 2019). Currently, it is not clear how PAR-calcium is delivered to the extracellular matrix, but it might be concentrated in extracellular vesicles involved in matrix mineralization (Muller et al., 2019).

The calcification of blood vessels is also initiated by the loss of calcification inhibitors [e.g., matrix-Gla protein (MGP), osteoprotegerin] (Luo et al., 1997; Bucay et al., 1998) or an imbalance in Pi/PPi/purine metabolism leading to a favorable environment for calcium phosphate deposition (Rutsch et al., 2021). Analysis of mineral phases on calcified aortas of MGP-deficient mice (*Mgp^–/–^*) using various spectroscopic and microscopic techniques suggest that calcification of elastin in the absence of MGP is reminiscent of multistep bone mineralization via amorphous calcium phosphate precursors to crystalline structures (hydroxyapatite) (Gourgas et al., 2018). Analysis of *Abcc6* and *Nt5e* knockout mice and mice lacking functional ectonucleotide pyrophosphatase/phosphodiesterase 1 (ENPP1) has led to the hypothesis that reduced plasma PPi, an inhibitor of calcium phosphate crystal growth, leads to a pro-mineralizing environment (Shimada et al., 2021). ABCC6 (ATP-binding cassette subfamily C member 6) facilitates cellular ATP efflux and the cascade of extracellular ATP degradation to adenosine involves hydrolyzes ATP to AMP and PPi by ENPP1. AMP is further hydrolyzed by CD73, a membrane-bound ecto-5′-nucleotidase encoded by *NT5E*, to generate adenosine and Pi. PPi is further hydrolyzed by tissue non-specific alkaline phosphatase (TNAP encoded by *ALPL*) to extracellular Pi. Adenosine is an indirect inhibitor of calcification by inhibiting TNAP (St Hilaire et al., 2011).

### Pathogenesis of Vascular Calcification in the Brain

Calcification of brain vessels is not specific to *Homo sapiens*, with calcification of cerebral arterioles, venules, and/or capillaries reported in laboratory mice, rats, domesticated animals (cats, dogs, cows, horses), and monkeys (Youssef et al., 2016). Only few studies have specifically characterized the pathomechanisms underlying brain vascular calcification and its associated changes (Table 1). It is likely that causes described for peripheral vessels are similarly involved in the calcification of cerebral vessels. However, vasculature is organotypic and recent advances in single cell RNA sequencing technology have generated insights into molecular differences and similarities between various vascular beds in the body (Augustin and Koh, 2017). Blood vessels in the body show different susceptibility to calcification, even in genetic diseases. For example, patients with mutations in gene *NT5E*, encoding for CD73, show preferential calcification of arteries in the extremities (St Hilaire et al., 2011; Zhang Z. et al., 2015). The cellular composition and characteristics of cerebral vessels differ from systemic vasculature. Cerebral endothelial cells possess the blood-brain barrier (BBB), a multicomponent feature that tightly controls parenchymal protein, metabolite, and ionic composition (Keller, 2013). In addition, other vascular cells (e.g., pericytes) have been shown to present organ-specific characteristics, at least at the level of the transcriptome (Vanlandewijck et al., 2018). Furthermore, blood vessels in the brain possess another characteristic not found in peripheral vessels: they are ensheathed with astrocyte end-feet, slender astrocyte processes, enriched with water and potassium channels (Schaffenrath and Keller, 2021). Microglial processes and the cell body contact the vessel basement membrane directly along the vascular tree (Mathiisen et al., 2010; Joost et al., 2019), thereby regulating blood flow in a purinergic receptor P2Y12-dependent manner (Bisht et al., 2021). Furthermore, the composition of the extracellular matrix is different along the vascular tree. In the case of medial calcification, the prevalent ECM component is elastin and elastin haploinsufficiency impedes the progression of arterial calcification (Khavandgar et al., 2014). Thus, given differences in cellular and acellular composition, the pathophysiology of vascular mineralization likely differs between arteries and capillaries.

**TABLE 1:** Alterations accompanying brain vascular calcifications.

Alteration/observation	Condition	Comment	References
Osteogenic environment	PFBC, AD, PD, aging	Accumulation of anti- and pro-calcification proteins in mineralized deposits on blood vessels	Fujita et al., 2003; Nahar et al., 2019; Zarb et al., 2019b
	PFBC	Cells expressing osteoblast markers around vascular calcifications.	Zarb et al., 2019b
	PFBC	Cells expressing osteoclast markers surrounding vascular calcifications. Osteoclast-like cells expressing cathepsin K are derived from microglia in a mouse model of PFBC.	Zarb et al., 2019b,^2021^
	Zika virus infection	Differentiation of Zika virus infected pericytes into osteoblast-like cells *in vitro* in response to BMP2.	Chen W. et al., 2021
Changes in the basement membrane	Aging	Electron microscopy studies reveal degeneration of vascular basement membrane and the presence of hydroxyapatite crystals in aged mice.	Morgan et al., 1982; Yanai et al., 1984
	Aging	Increased deposition of collagen I in vascular basement membrane in aged mice.	Yang et al., 2020
	HCHWA-D	Vascular mineralization is preceded by accumulation of osteopontin and the appearance of fibrotic collagen I in autopsy samples.	Grand Moursel et al., 2019
Oxidative stress	PFBC	Accumulation of 2-ω-carboxyethylpyrrole CEP adducts in astrocytes surrounding vascular calcifications in a mouse model of PFBC.	Zarb et al., 2019b
Altered levels of inorganic phosphate	PFBC	Higher inorganic phosphate levels in the CSF in *Slc20a2^–/–^* mice and *SLC20A2* mutation carriers	Jensen et al., 2016; Paucar et al., 2017; Wallingford et al., 2017; Hozumi et al., 2018
	Aging	Endothelial cells of old mice are expressing higher levels of TNAP	Yang et al., 2020

*AD, Alzheimer’s disease; HCHWA-D, hereditary cerebral hemorrhage with amyloidosis-Dutch type; PD, Parkinson’s disease; PFBC, primary familial brain calcification.*

Vascular calcification increases with age but histopathological studies describing vascular calcifications in the aged human brain are rare (Fujita et al., 2003; Peters et al., 2018; de Brouwer et al., 2021). In general, arterial and capillary calcifications in aged brains have similar histological characteristics as described in various brain pathologies. In aged mice, thalamic blood vessels show the tendency to calcify (Fraser, 1968; Morgan et al., 1982; Yanai et al., 1984, 1987). A recent study showed that vessel-associated calcifications in the thalamus in old wild-type mice show an affinity for bone labeling dyes (Alizarin red and Osteosense). Additionally, aged endothelial cells showed increased TNAP (generates Pi) expression and collagen I deposition in the vascular basement membrane (Yang et al., 2020). The question of whether these matrix mineralization-associated proteins triggered thalamic vessel calcification was not investigated. Although no inflammatory reaction toward aging-related vessel calcifications was described using transmission electron microscopy (TEM) (Morgan et al., 1982), additional studies using other methods (e.g., immunohistochemistry) are needed to investigate whether age-related calcifications evoke a glial response. TEM studies on vascular calcifications in monkey and mouse brains have described early changes in the basement membrane with crystalline hydroxyapatite deposits coinciding with cellular debris or basement membrane degeneration (Morgan et al., 1982; Yanai et al., 1984, 1987, 1994). Alterations in the composition of the basement membrane (accumulation of osteopontin and the appearance of fibrotic collagen I) have also been reported to precede matrix mineralization in the case of hereditary cerebral hemorrhage with Dutch type -amyloidosis (Grand Moursel et al., 2019). Age is an independent risk factor for both the development of cardiovascular disease and neurodegenerative disease (Yazdanyar and Newman, 2009; Hou et al., 2019), and thus, more studies are needed to characterize changes in aged blood vessels that lead to vascular calcifications in the brain.

In order to describe the pathophysiology of brain vessel calcification in detail, we will refer to studies investigating vascular calcification in PFBC.

#### Primary Familial Brain Calcification

As mentioned above, the list of diseases with brain calcification as a secondary manifestation is extensive and it has not been intensively investigated whether these calcifications are vascular and/or parenchymal. In the case of hereditary neuropsychiatric disease, primary familial brain calcification (PFBC), the presence of bilateral basal ganglia vascular calcifications is a diagnostic criterion (Balck et al., 2021). PFBC patients may present clinically with motor (e.g., parkinsonism, ataxia, speech disturbance) and/or non-motor (cognitive deficit, depression, psychosis) phenotypes or may even remain unaffected (Balck et al., 2021). In PFBC, the capillary bed is encrusted with the calcium phosphate deposits like “pearls on the string” in addition to the calcification of the medial layer of arteries and arterioles (Miklossy et al., 2005; Kimura et al., 2016). These small, mineralized deposits are located on the capillary wall adjacent to the parenchyma (Kobayashi et al., 1987) (Figures 1A,D).

Autosomal-dominant (AD)- PFBC is caused by mutations in the platelet-derived growth factor subunit B *(PDGFB)* (Keller et al., 2013) and its receptor–*PDGFRB* (Nicolas et al., 2013b), solute carrier family 20 member 2 *(SLC20A2)* (Wang et al., 2012), and xenotropic and polytropic retrovirus receptor 1 *(XPR1)* (Legati et al., 2015). SLC20A2 (also known as PiT2) is an inorganic phosphate importer (Kavanaugh et al., 1994) and XPR1 is the only known mammalian inorganic phosphate exporter (Giovannini et al., 2013). PDGFB and PDGFRB are growth factor and receptor, respectively, implicated in organ development (Andrae et al., 2008; Armulik et al., 2011). Autosomal-recessive (AR) form of PFBC is caused by mutations in myogenesis regulating glycosidase (*MYORG*) (Yao et al., 2018) and junctional-adhesion-molecule-2 (*JAM2*) (Cen et al., 2020). The cellular function of MYORG, an intracellular transmembrane protein belonging to glycosyl hydrolase family, is currently unknown. The knock-out of *Myorg* (alternative name in the mouse genome is AI464131) in mice does not lead to gross developmental defects (Yao et al., 2018). *JAM2* encodes for the transmembrane protein located to cell-cell adhesions and is implicated in trans-endothelial migration of leukocytes (Aurrand-Lions et al., 2001). The genes that cause both, AD-PFBC and AR-PFBC, are functionally different, and expressed by several cell types in the brain (Zarb et al., 2019a). XPR1 and SLC20A2 are ubiquitously expressed (Zeisel et al., 2018). PDGFRB is expressed by perivascular fibroblasts, vascular smooth muscle cells, pericytes and, astrocytes. PDGFB, the main ligand for PDGFRB, is expressed by endothelial cells and microglia, and by certain excitatory and cholinergic neurons (Armulik et al., 2011; Vanlandewijck et al., 2018; Zeisel et al., 2018). MYORG is expressed only by astrocytes (Yao et al., 2018) and JAM2 by perivascular fibroblasts, endothelial cells, and astrocytes (Vanlandewijck et al., 2018). Recent investigations using animal models and genetic analyses have led to a better understanding of the pathophysiology of vascular calcification in PFBC. Currently, three mouse models mimic the histopathological features of PFBC: *Slc20a2* (Jensen et al., 2013) and *Myorg* (Yao et al., 2018) knockouts, and PDGFB hypomorph (*Pdgfb^ret/ret^*, retention motif knockout) (Keller et al., 2013). The retention motif knockout renders a biologically active PDGFB protein unable to bind extracellular heparan sulfate proteoglycans and thus, shape PDGFB gradients (Abramsson et al., 2003; Lindblom et al., 2003). In PFBC mutation carriers, calcification of the medial layer of arteries, as well as capillaries, is observed (Kozik and Kulczycki, 1978; Miklossy et al., 2005; Kimura et al., 2016). In addition, calcium precipitates have been reported on neurons and astrocytes (Kobayashi et al., 1987; Miklossy et al., 2005). Vascular calcifications are protein-rich and contain hydroxyapatite and trace elements (Zn, Fe) (Smeyers-Verbeke et al., 1975; Bouras et al., 1996). Vascular calcification is similar in mouse models of PFBC with single rounded or mulberry shaped lamellar structures deposited on the vessel wall of arterioles and capillaries and protruding into the parenchyma (Jensen et al., 2013; Keller et al., 2013) (Figure 1). Calcifications appear as nodules such that the entire capillary is occasionally surrounded by ring-like calcification. These calcifications contain calcium and phosphate as well as collagenous and non-collagenous bone proteins. They bind bisphosphonates, are of bone density and stain for histological dyes used to visualize bone (e.g., Alizarin red, Alcian blue) (Jensen et al., 2013, 2018; Keller et al., 2013; Yao et al., 2018; Zarb et al., 2019b). Mass spectrometry has been used to characterize calcifications in mice and has shown that they contain proteins that promote (e.g., secreted glycoprotein SPARCL1) or halt calcification (fetuin A, MGP, osteopontin) (Nahar et al., 2019). In addition, they contain amyloid precursor protein (APP), amyloid precursor-like protein 2 (APLP2), as well as secretogranin-1 (CHGB) and chromogranin A (CHGA), which are major constituents of large dense core vesicles involved in storing and delivering large neurotransmitters in neurons (Nahar et al., 2019). Accordingly, these calcifications can be visualized in tissue sections using antibodies against APP, APLP2, osteopontin, osteocalcin, collagen I (Nahar et al., 2019; Zarb et al., 2019b,^2021^). Although calcifications contain APP, APLP2 and aggregated protein structures, these structures lack the β-pleated sheet conformation and structural regularity recognized by Thioflavin T or Congo red (Zarb et al., 2021). Chromogranins (CHGB, CHGA) are deposited in the amyloid plaques in AD patients and are deregulated in other brain disorders such as multiple sclerosis, amyotrophic lateral sclerosis, schizophrenia (Willis et al., 2011). Interestingly, a recent study identified a chromogranin A derived peptide from the adrenal gland, which inhibits the osteogenic trans-differentiation of VSMC *in vitro* (Orth-Alampour et al., 2021).

The underlying mechanism leading to vascular calcification and constituent cell types is currently unclear. The genes that cause both AD-PFBC and AR-PFBC are functionally different and expressed by several cell types at the neurovascular unit. The pathogenesis of PFBC may be multifactorial with mutations potentially affecting multiple cell types in brain vessels and leading to the formation of vessel-associated calcifications. Cell type specific knockouts of involved genes should clarify the role of individual cell types. However, it has been demonstrated in mice that the endothelial expression of PDGFB is protective against brain calcifications (Keller et al., 2013), indicating that vessel-calcification might be caused by reduced PDGFB/PDGFRB signaling at the vessel wall. In summary, it remains to be discovered why mutations in structurally and functionally different protein families lead to the same disease and calcifications of cerebral arteries and capillaries.

##### Does Imbalance in Brain Phosphate Metabolism Cause Vascular Calcification?

One hypothesis is that vascular calcification in PFBC is caused by the locally altered phosphate levels in the cerebrospinal fluid (CSF) and perivascular spaces (Wallingford et al., 2017). There are no abnormalities in phosphate and calcium levels in the serum of PFBC patients (Manyam, 2005) and PFBC animal models (Keller et al., 2013; Jensen et al., 2016). In addition, analysis of serum calcification propensity of *Pdgfb^ret/ret^* mice did not point to alterations in the humoral anti-calcification defense (Zarb et al., 2019b). Studies on *Slc20a2*^–/–^ mice suggest that loss of this phosphate importer increases local phosphate concentration in the cerebrospinal fluid, leading to calcium-phosphate deposition in the glymphatic space due to imbalanced phosphate metabolism and subsequent calcification of arterioles (Wallingford et al., 2017). Also, *SLC20A2* (but not *PDGFB*) mutation carriers show elevated levels of Pi in CSF (Jensen et al., 2016; Paucar et al., 2017; Wallingford et al., 2017; Hozumi et al., 2018). It was proposed that changes in Pi concentrations in the CSF could be caused by altered Pi absorption by choroid plexus epithelial cells (Wallingford et al., 2017). SLC20A2 belongs to the type III family of inorganic phosphate importers that consists of two proteins—SLC20A1 and SLC20A2 (frequently used protein name is PiT1 and PiT2, respectively). These proteins are known to maintain cellular phosphate homeostasis (Lederer and Miyamoto, 2012). Therefore, it is difficult to envision how *SLC20A2* mutations could lead to changes in the extracellular phosphate levels.

Cellular ATP and inorganic phosphate levels are sensed by cells by detecting changes in inositol pyrophosphate concentration (Azevedo and Saiardi, 2017). Previous studies have shown that XPR1 senses the intracellular phosphate level by binding to inositol pyrophosphates via its SPX domain (Wilson et al., 2019; Li et al., 2020). Proteins regulating various aspects of phosphate metabolism accomplish this via the SPX (named after yeast proteins Syg1, Pho81 and the mammalian Xpr1) domain. Yeast and plants have several SPX-domain-containing proteins but mammals have only one protein – XPR1 (Azevedo and Saiardi, 2017). A recent study identified crosstalk between SLC20A2 and XPR1 for maintaining constant intracellular phosphate and ATP levels, where XPR1 is a key inositol pyrophosphate-dependent regulator of this process (Lopez-Sanchez et al., 2020). An electron microscope study on *Slc20a2*^–/–^ mice showed intracellular calcium phosphate crystals in pericytes and astrocytes (Jensen et al., 2018), indicating that the initial crystallization of calcium phosphate could occur intracellularly. Thus, the XPR1 and SLC20A2 mutation carriers could show altered intracellular phosphate metabolism. The consequences on vascular cells and how this leads to vascular calcification remain to be determined.

##### Blood-Brain Barrier Dysfunction–A Cause for Vascular Calcification in Primary Familial Brain Calcification?

Histopathological changes such as the extravasation of plasma proteins observed in the PFBC autopsy cases and the presence of vasogenic edema detected by magnetic resonance imaging indicate BBB dysfunction (Gomez et al., 1989; Miklossy et al., 2005). The initial interpretation of data suggested pericyte deficiency and BBB deficiency in PFBC as a potential link between the formation of brain calcifications (Keller et al., 2013). However, later studies on mouse models did not find evidence to support this view. The brain regions associated with vascular calcification (i.e., thalamus, mesencephalon, and dorsal pons) in *Pdgfb^ret/ret^* mice showed significantly lower BBB permeability (Vanlandewijck et al., 2015). It is likely that BBB permeability changes in PFBC occur independently and are not a direct cause of vascular calcification. No BBB disruption has been detected in *Slc20a2^–/–^* mice (Wallingford et al., 2017; Nahar et al., 2019). Interestingly, gene mutations in endothelial junctional proteins occludin, junctional adhesion molecule 3 (JAM3) and cerebral cavernous malformation (CCM) −1, −2, −3 lead to the BBB deficiency and brain calcification (Saitou et al., 2000; Mochida et al., 2010; O’driscoll et al., 2010; Fischer et al., 2013). In addition, biallelic-mutations in a gene encoding a cell-cell junction protein JAM2 causes PFBC (Cen et al., 2020; Schottlaender et al., 2020). This suggests that specific alterations in cell-cell adhesion at the vascular wall could lead to vascular calcification, which are not necessarily dependent on BBB permeability.

##### Phenotypic Change of Vascular Cells to Osteoblast-Like Cells in Primary Familial Brain Calcification?

The PDGFB/PDGFRB pathway is crucial for pericyte development (Armulik et al., 2011), which raises the question as to whether vascular calcification in PDGFB and PDGFRB mutation carriers is caused by the lack of pericytes. As discussed above, follow-up studies have not supported pericyte deficiency as a cause of vascular calcification in PDGFB mouse model (Vanlandewijck et al., 2015). In *Pdgfb^ret/ret^* mice, the vessels in deep brain regions had a higher pericyte coverage and less BBB leakage compared to cortical vessels, a region that does not calcify (Vanlandewijck et al., 2015). Thus, one might speculate that the presence of pericytes is needed for the development of calcifications. In addition, an alteration in pericyte numbers has not been observed in *Slc20a2*^–/–^ mice (Wallingford et al., 2017; Nahar et al., 2019). Characterization of the extracellular environment surrounding vascular calcifications showed a so-called osteogenic environment in *Pdgfb^ret/ret^* mice and human PFBC cases (Zarb et al., 2019b). Calcifications were associated with cells expressing markers of osteoblasts, and osteocytes (Zarb et al., 2019b), but the cellular origin of these cells is not known. Cultured pericytes, depending on culture conditions, have the capacity to form extracellular calcifications containing hydroxyapatite (Schor et al., 1990). Interestingly, *in vitro* infection of human fetal pericytes with Asian strain of Zika virus led to dedifferentiation of pericytes into bone forming cells via upregulation of bone morphogenetic protein 2 (BMP2) (Chen W. et al., 2021). Also, endothelial cells have the capacity to give rise to bone-forming cells, as was shown in fibrodysplasia ossificans progressiva, a disease characterized by extensive extraskeletal bone formation (Medici et al., 2010; Yao et al., 2013). Curiously, astrocytes have also been linked to the formation of chondrocytes in human gliomas in which a gradual morphologic change of astrocytes to cells indistinguishable from chondrocytes was observed (Kepes et al., 1984). Phenotypic alterations in pericytes in mouse models of PFBC have not been extensively investigated. Isolated brain vessels in PDGFB hypomorphs show upregulation of *Bmp2* and *Bmp4* expression (Armulik et al., 2010). Single cell RNA sequencing of brain endothelial cells showed that both *Bmp2* and *Bmp4* expression is elevated in *Pdgfb^ret/ret^* mice (Mae et al., 2021). *Bmps* are expressed by normal endothelial cells and pericytes. Altered *Bmp* expression in *Pdgfb^ret/ret^* mice could reflect alterations caused by disturbed pericyte-endothelial crosstalk due to reduced pericyte numbers (Armulik et al., 2010; Vanlandewijck et al., 2018; Mae et al., 2021). Upregulated BMP signaling induces proinflammatory effects in endothelial cells (Cai et al., 2012). Aortic calcification is known to be associated with inflammation and dependent on BMP2 signaling (Canet-Soulas et al., 2021). It remains to be investigated whether deregulated expression of *Bmp2* and *Bmp4* contributes to vascular inflammation and calcification in *Pdgfb^ret/ret^* mice (Torok et al., 2021).

##### What Is the Role of Astrocytes in Brain Vascular Calcification?

Vessel associated calcifications elicit a conspicuous glial response in mouse models and human PFBC cases (Chakrabarty et al., 2011; Keller et al., 2013; Zarb et al., 2019b) (Figure 1). TEM images reveal that calcifications in mouse models of PFBC are surrounded by glia – either astrocytes or microglia (Nahar et al., 2019). Microglia surrounding calcifications contain inclusions of phagocytosed material, whereas astrocytes surrounding calcifications show signs of degeneration and the accumulation of calcium crystals (Jensen et al., 2018; Nahar et al., 2019). Strongly glial fibrillary acidic protein (GFAP) positive astrocytes surrounding calcifications express podoplanin (Zarb et al., 2019b), a protein strongly expressed by a subset of reactive astrocytes in glioblastoma, and after ischemic and stab wound injuries (Kolar et al., 2015). In addition, astrocytes surrounding calcifications also express proteins like complement 3 and lipocalin 2, which are commonly upregulated during various insults (Bi et al., 2013). Astrocytes around calcifications could potentially lead to neuronal damage and modify the inflammatory response. In addition, GFAP-positive astrocytes around calcifications are positive for 2-ω-carboxyethylpyrrole (CEP) adducts, a peroxidation product of docosahexaenoic acid (Zarb et al., 2019b). It remains to be shown whether the CEP is produced in astrocytes surrounding calcifications or scavenged from the environment. Nevertheless, the accumulation of CEP is characteristic of inflammation-associated oxidative stress in the human brain (Xiong et al., 2021), which indicates the presence of oxidative stress, a well-characterized stimulator and accelerator of vascular calcification (Pescatore et al., 2019), in PFBC. Thus, accumulating evidence indicates that astrocytes could play a role in PFBC pathology. Interestingly, MYORG, a gene mutated in AR-PFBC, is exclusively expressed by S100β positive astrocytes in the mouse brain (Yao et al., 2018). This observation raises the question of whether cell-autonomous changes in astrocytes lead to vascular calcification. In fact, astrocytes are the only cell type at the neurovascular unit expressing all genes, except the PDGFB, causing PFBC.

### Microgliopathies and Brain Calcification

Microglia, resident tissue macrophages in the brain, are essential for the proper central nervous system (CNS) development and recovery from injury (e.g., remyelination) (Li and Barres, 2018). In addition, microglia are responsible for sensing and removing self-injurious proteins such as aggregated Aβ, α-synuclein, and prions (Hickman et al., 2018). Many AD risk genes are expressed by microglia and are involved in modifying microglial phagocytosis among other pathways (Hansen et al., 2018). Brain calcifications comprise one of the pathological hallmarks of a wide range of brain diseases affecting microglia or causing autoinflammation. Patients with mutations in genes implicated in microglial development and function [e.g., interferon regulatory factor 8 (*IRF8*), colony stimulating factor 1 receptor (*CSF1R*), triggering receptor expressed on myeloid cells 2 (*TREM2*), negative regulator of reactive oxygen species (*NRROS*)] harbor intracerebral calcifications (Rademakers et al., 2011; Konno et al., 2017; Bigley et al., 2018; Dong et al., 2020; Chitu et al., 2021). Patients with Nasu-Hakola disease, a neurodegenerative disease caused by mutations in TYRO protein tyrosine kinase-binding protein (*TYROBP*, also known as DAP12) or *TREM2*, present with cerebrovascular changes and bilateral basal ganglia calcifications localized to the walls of blood vessels (Kalimo et al., 1994; Coomans et al., 2018). Of note, brain calcification has not yet been reported in various mouse *Csf1r* mutants or in mice deficient of *Trem2*, *Dap12*, *Lrrc33* (NRROS) and *Irf8* but it would be of great interest to investigate whether these mice develop calcifications during aging or along with existing pathologies. However, long-term elimination of microglia using the CSF1R inhibitor, PLX5622, in control and PFBC mice resulted in localized axonal damage to fiber tracts of the internal capsule and adjacent thalamic and striatal areas, characterized by bone protein containing axonal spheroids (Zarb et al., 2021). This indicates that the chronic absence of CSF1R signaling could also lead to white matter calcification in mice.

Dysregulation of the type I interferon pathway, an essential component of the brain’s innate immune defense, also triggers brain calcification, which is considered a useful radiological imaging marker for these diseases (Mcglasson et al., 2015). Mutations in ubiquitin-specific peptidase 18 (*USP18*), a ubiquitin-specific protease, which negatively regulates type I interferon-signaling, lead to brain calcifications in humans (Meuwissen et al., 2016). Microglia specific deletion of *Usp18* in mice leads to the formation of calcifications (Goldmann et al., 2015). On the other hand, a baseline type I interferon signaling in microglia is necessary to prevent calcification. Immunodeficiency caused by interferon-stimulated gene 15 (*IGS15*) mutations leads to intracranial calcifications in humans (Zhang X. et al., 2015).

Although brain calcification is a pathological hallmark of the above-mentioned diseases, the pathogenesis and the role of microglial involvement has not received much attention. One of the reasons could be that calcifications in brain tissue are generally considered clinically irrelevant due to their occurrence in aged individuals (Arnold and Kreel, 1991). Nevertheless, the presence of calcifications is an indication of altered homeostasis and diseases caused by a cell-autonomous defect in microglia are associated with brain calcification. Thus, microglial function seems to be critical in controlling brain calcification, either by removing apoptotic cells or controlling proteostasis of the ECM, and thereby nucleation of calcium phosphate, and/or removal of hydroxyapatite. Babies born with congenital Zika virus infection have intracerebral calcifications at birth (Moore et al., 2017), but show progressive clearance of intraparenchymal calcifications (Petribu et al., 2017), suggesting that calcifications can be reabsorbed. This raises the question of how and which cells are responsible for removing calcifications. It would not be farfetched to suggest that microglial activity plays a cardinal role.

#### Microglia and Vascular Calcifications in Primary Familial Brain Calcification

**FIGURE 2:**
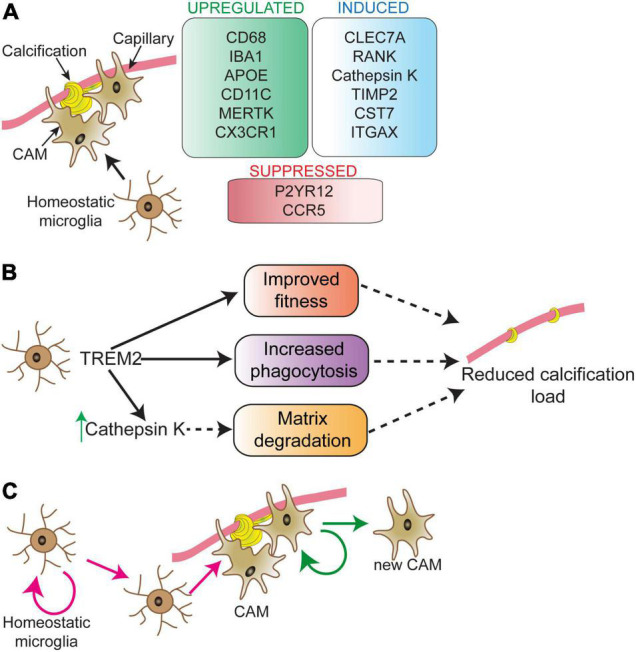
Microglial phenotype in response to vascular calcifications. Calcification-associated microglia (CAM) show a distinct molecular signature **(A)**. Signature changes partially overlap with disease-associated microglia (DAM), except for RANK and cathepsin K expression, which are unique to CAM. Modifying microglial activity via TREM2 can play a direct role in controlling vessel calcification load **(B)**. The process through which TREM2 activity controls calcification load is still unknown. TREM2 could sustain microglial fitness and support microglial phagocytosis resulting in reduced calcification load. TREM2 activity is upstream of cathepsin K. Proliferating microglia surround vascular calcifications **(C)**. The nature of proliferating microglia in the near vicinity of calcifications and their fate is not well-understood. Depicted are two possibilities (pink and green arrows) for the origin and fate of proliferating microglia surrounding vessel calcifications. These possibilities are: expansion of calcification-associated microglia (green arrows) and/or replacement of parenchymal microglia in the vicinity of calcified vessels (pink arrows). CAM, calcification-associated microglia.

Microglia surround brain calcifications in human PFBC cases and PFBC mouse models (Miklossy et al., 2005; Nahar et al., 2019; Zarb et al., 2019b,^2021^). A recent study using the PFBC mouse model (*Pdgfb^ret/ret^* mice) presented evidence that microglia play a protective role by halting the calcification of capillaries (Zarb et al., 2021). Transcriptomic analysis of calcified mouse brain tissue showed deregulation of inflammatory pathways associated with activated microglia. Microglia acquire various insult-dependent activation profiles, however, some of the signature genes are shared by different insults (Candlish and Hefendehl, 2021). While the expression of many proteins [e.g., lipoprotein lipase (LPL), CD68, integrin alpha X (ITGAX), cystatin F (CST7), C-Type Lectin Domain Containing 7A (CLEC7A or dectin-1)] by microglia surrounding calcifications or calcification-associated microglia (CAM) is shared with the previously identified core signature (Keren-Shaul et al., 2017), there are differences in the expression profile (e.g., expression of cathepsin K) (Figure 2A). Lineage tracing experiments have shown that cathepsin K expressing cells are derived from microglia (Zarb et al., 2021). In bone, cathepsin K, the principal collagen I degrading enzyme, is secreted by osteoclasts (Drake et al., 1996; Costa et al., 2011). In addition to cathepsin K, microglia surrounding calcifications express receptor activator of nuclear factor κ B (RANK or TNFRSF11A) (Zarb et al., 2021), a receptor known to regulate osteoclast differentiation (Li et al., 2000). Thus, microglia surrounding calcifications might acquire an osteoclast-like phenotype necessary for removing the calcified extracellular matrix. Interestingly, osteoclasts and brain macrophages share key signaling pathways for their development (e.g., TREM2/DAP12, CSF1) (Lee et al., 2021). During embryogenesis, osteoclasts originate from erythro-myeloid progenitors in the embryo and are subsequently maintained in adulthood by hematopoietic stem cells-derived blood leukocytes (Jacome-Galarza et al., 2019). Alternatively, microglia originate from erythro-myeloid precursors in the yolk sac (Ginhoux et al., 2010; Schulz et al., 2012; Goldmann et al., 2016; Utz et al., 2020) and are maintained by self-renewal in the adult. The brain harbors several macrophage populations: parenchymal macrophages (i.e., microglia) and non-parenchymal macrophages, which reside at CNS border regions including the meninges, choroid plexus and perivascular spaces (Kierdorf et al., 2019). Further studies using Cre-lines that distinguish microglia from perivascular macrophages, and peripheral myeloid cells are needed to clarify whether cathepsin K expressing cells surrounding vessel calcifications are derived exclusively from microglia. In addition, it remains to be investigated whether brain perivascular macrophages and/or peripheral monocytes modify cerebral vascular calcification. Atherosclerotic lesions contain several macrophage populations, which have different capacities to promote or halt lesion development (Willemsen and De Winther, 2020). Although macrophages in these lesions express osteoclast markers (tartrate-resistant acid phosphatase, cathepsin K, carbonic anhydrase 2), they have low mineral resorption potential due to altered nuclear factor of activated T cells type c-1 (NFATC1) signaling, resulting in a lower expression and activity of cathepsin K (Chinetti-Gbaguidi et al., 2017). Calcification-associated microglia specific knockout of cathepsin K should clarify its importance in degrading vascular calcifications in the PFBC (Figure 2B). Of note, there is also heterogeneity within calcification-associated microglia in PFBC. The expression of markers such as Ionized calcium binding adaptor molecule 1(IBA1) and CLEC7A are detected in all microglia surrounding calcifications, whereas expression of some markers (e.g., cathepsin K, ITGAX) are detected only in a subset of microglia (Zarb et al., 2021).

Long-term removal of microglia using the CSF1R inhibitor PLX5622 resulted in an increase in vessel-associated calcification load in *Pdgfb^ret/ret^* mice. Similar results were obtained in *Pdgfb^ret/ret^* mice lacking one or two functional alleles of *Trem2* (Zarb et al., 2021). The absence of cathepsin K expression by microglia surrounding calcifications in these mice indicates that TREM2 activity could be necessary to induce osteoclast-like phenotype in microglia (Figure 2B). Interestingly, single cell analysis of atherosclerotic lesions isolated from mouse models of atherosclerosis showed the presence of TREM2^high^ macrophages, which were enriched for genes highly expressed by osteoclasts, suggesting a role in lesion calcification (Cochain et al., 2018). This population also expressed markers for so-called disease-associated microglia described in aging and mouse models of AD (Keren-Shaul et al., 2017). It remains to be investigated whether the expression of osteoclast genes in TREM2^high^ macrophages is TREM2 dependent and whether these cells modify the calcification of atherosclerotic lesions.

Microglia turnover rate in the normal brain is relatively low, but under pathological conditions, the microglial turnover rate is increased (Prinz et al., 2019). Accordingly, conspicuous microglial proliferation can be detected in brain regions that develop vascular calcifications in a mouse model of PFBC (Zarb et al., 2021). There might be a higher microglial turnover rate in these regions since phagocytosis/degradation of calcifications by microglia could lead to exhaustion and death, similar to the microglial dynamics detected in the mouse model of AD (Prinz and Priller, 2017). It needs to be investigated whether there is a clonal expansion of CAM (Figure 2C, green arrows) and/or replacement of parenchymal microglia in the vicinity of calcified vessels which give rise to CAM (Figure 2C, pink arrows).

Microglia have emerged as disease modifiers in a wide range of neurodegenerative diseases (e.g., AD) (Hickman et al., 2018). Of six genes implicated in PFBC (*JAM2*, *PDGFB, PDGFRB, SLC20A2, XPR1, MYORG*), microglia express *JAM2, PDGFB, SLC20A2, XPR1*. Microglia do not express the receptor—PDGFRB, and thus, it is unlikely that *PDGFB* or *PDGFRB* haploinsufficiency in PFBC causes cell-autonomous microglial dysfunction. Nevertheless, cells of monocyte origin derived from PDGFB hypomorphs and PFBC patients carrying PDGFB mutation were reported to show defects in osteoclast-differentiation *in vitro* (Schiemenz et al., 2020). However, whether this finding also applies to brain macrophages was not investigated. Interestingly, *xpr1b*, an orthologue of *XPR1*, is crucial for the differentiation of tissue-resident macrophages and microglia in zebrafish (Meireles et al., 2014), indicating that PFBC genes could modify the development of tissue resident macrophages.

Currently, it is not known how microglia sense and degrade calcifications. Although microglia surround and phagocytose calcifications, there is currently no understanding of how they sense calcifications on vessels. Future studies should clarify how the calcified extracellular matrix on the vessel wall is sensed by microglia. In general, little is known how extracellular calcium phosphate crystals are recognized by macrophages (Mulay and Anders, 2016). It could involve various pattern-recognition receptors (PRRs) implicated in recognition damage- or pathogen-associated patterns. Recently, CLEC12A, a PRR belonging to the same class as CLEC7A, induced in CAM, was shown to recognize inflammation causing deposition of uric acid crystals in joints in gout (Neumann et al., 2014). However, this interaction was shown to be necessary to inhibit the immune response, as mice deficient in *Clec12a* exhibited hyperinflammation in response to uric acid crystals (Neumann et al., 2014). Another possibility is that the recognition/uptake of calcifications is receptor-independent and mediated via membrane lipids, similar to solid structure uptake in dendritic cells (Ng et al., 2008). Stiffness of vascular walls most likely differs between normal and calcified vessels such that microglia could sense and respond to altered tissue stiffness. Microglia have the capacity to respond to tissue stiffness as in the retina, where microglial phenotype and the control of vascular architecture was dependent on tissue stiffness and regulated by integrin signaling (Dudiki et al., 2020). As has been shown in other types of vascular injury, microglia could also respond to vascular calcifications by sensing alterations via purinergic receptor P2Y12 (Lou et al., 2016). Although it is not known whether vascular calcification in the brain involves alterations in purinergic signaling, altered Pi/PPi/purinergic signaling has been shown to cause vascular calcification in pseudoxanthoma elasticum (Rutsch et al., 2021). Additionally, it would be necessary to dissect signaling pathways that could trigger a coordinated degradation of proteins and hydroxyapatite by microglia. As discussed above, TREM2 activity is essential but most likely additional signaling pathways participate in this process. Since macrophages are capable of degrading phagocytosed hydroxyapatite (Hyvonen and Kowolik, 1992), it would be interesting to investigate whether microglia show differential capacity to degrade matrix depending on the Ca-phosphate phases (i.e., amorphous calcium phosphate vs hydroxyapatite).

## Outlook

Brain vessel calcifications that accompany aging are shared by diverse brain pathologies, and in some cases, comprise a diagnostic criterion. The brain possesses an energy reserve limited to only a few minutes, and therefore, brain function is dependent on continuous blood flow delivered by the vasculature (Mergenthaler et al., 2013; Iadecola, 2017). Although vascular calcification is unlikely to be beneficial, insights into the consequences of vessel calcification on neurovascular coupling and brain function are needed. In addition, it is important to understand the vascular and parenchymal alterations preceding mineralization of the vessel wall as well as longitudinal changes in the composition of mineral phases and other components such as proteins and metabolic ions. Accumulating evidence suggests that dysfunction of resident brain macrophages leads to tissue calcification. Thus, removal of calcium phosphate precipitates in the brain parenchyma and vessel wall could be considered as a part of microglia parenchymal surveillance along with other well-described functions. However, the direct function of CAM is currently unclear and requires further study. Also, excellent questions for future research are how microglia recognize and degrade vascular calcifications and how to therapeutically target microglia in order to reduce vascular calcification.

## Author Contributions

AK wrote the first draft of the manuscript. All authors contributed to manuscript revision, read, and approved the submitted version.

## Conflict of Interest

The authors declare that the research was conducted in the absence of any commercial or financial relationships that could be construed as a potential conflict of interest.

## Publisher’s Note

All claims expressed in this article are solely those of the authors and do not necessarily represent those of their affiliated organizations, or those of the publisher, the editors and the reviewers. Any product that may be evaluated in this article, or claim that may be made by its manufacturer, is not guaranteed or endorsed by the publisher.
